# Glucocorticoids and immunoglobulin alone or in combination in the treatment of multisystemic inflammatory syndrome in children: a systematic review and network meta-analysis

**DOI:** 10.3389/fped.2025.1545788

**Published:** 2025-05-29

**Authors:** Junjie Lin, Qiang Tong, Hui Huang, Jiahui Liu, Ye Kang, Siyuan Liu, Weiyi Wang, Tianshu Ren, Yuan Yuan

**Affiliations:** ^1^ Department of Pharmacy, General Hospital of Northern Theater Command, Shenyang, China; ^2^ Department of Clinical Pharmacy, Shenyang Pharmaceutical University, Shenyang, China

**Keywords:** intravenous immunoglobulin, glucocorticoids, multisystemic inflammatory syndrome in children, COVID-19, combination therapy

## Abstract

**Importance/background:**

Multisystem Inflammatory Syndrome in Children (MIS-C) has sparked the creation of diverse treatment guidelines by healthcare organizations globally. The initial management strategies for MIS-C differ among these guidelines. In developed nations, intravenous immunoglobulin (IVIG) is frequently advised as the first-line treatment. However, given its high cost and limited availability in numerous countries, there is a pressing need for evidence to validate alternative therapeutic options.

**Objective:**

To evaluate the efficacy of glucocorticoids (GCs), IVIG, and combination therapy for the treatment of Children with MIS-C.

**Data sources:**

PubMed, Cochrane Library, EMBASE, Web of Science, and Medellín. The last search update was on April 8, 2025.

**Data extraction and synthesis:**

Cohort studies that evaluated the efficacy of IVIG, GCs, and IVIG combined with GCs in children clinically diagnosed with MIS-C were included. Two authors independently screened the studies, extracted relevant data, and assessed the risk of bias.

**Primary outcomes and measures:**

The primary outcomes of the Bayesian network meta-analysis were inotropic support requirements, treatment failure/persistent fever, left ventricular (LV) dysfunction, need for adjuvant immunotherapy, mortality and coronary artery dilatation/aneurysm. Secondary outcomes included length of stay in the intensive care unit (ICU), duration of fever, and duration of inotropic support.

**Results:**

The primary analysis included fourteen cohort studies with a total of 4,269 participants. According to moderate-quality evidence, combination therapy demonstrated the most significant reduction in the need for adjuvant immunotherapy compared to IVIG alone [OR 0.29, 95% CI (0.19, 0.45)]. Additionally, GCs monotherapy was found to be most effective in lowering the incidence of treatment failure [OR 0.23, 95% CI (0.14, 0.39)]. When compared to combination therapy, GCs monotherapy was associated with a reduction in ICU length of stay [SMD −0.25, 95% CI (−0.85, 0.36)], duration of fever (SMD [−0.42, 95% CI (−0.73, −0.11)], and duration of inotropic support [SMD −0.13, 95% CI (−0.46, 0.20)], as well as a decrease in the incidence of left ventricular (LV) dysfunction [OR 0.96, 95% CI (0.55, 1.68)]. Furthermore, GCs monotherapy had the lowest incidence of coronary artery dilation/aneurysm, while combination therapy required the least inotropic support. Patients receiving IVIG had the lowest mortality rate, but no statistically significant mortality differences existed across treatment groups.

**Conclusions and relevance:**

GCs monotherapy significantly reduces treatment failure rates and persistent fever duration, while combination therapy significantly reduces the need for adjunctive immunotherapy. For countries with limited access to IVIG, initiating GCs as first-line therapy may be a viable option.

**Systematic Review Registration:**

https://www.crd.york.ac.uk/PROSPERO/, PROSPERO identifier CRD42023456156.

## 1 Introduction

In 2020, amid the global COVID-19 pandemic, medical facilities worldwide began reporting cases of children and adolescents presenting with multisystem inflammation linked to SARS-CoV-2 infection. In April of the same year, the United States Centers for Disease Control and Prevention (CDC) designated this condition as MIS-C. Due to its rapid progression, which can lead to critical illness, shock, multiple organ failure, and even death, over 80% of affected children require intensive care unit (ICU) admission for treatment. The clinical presentation often resembles that of Kawasaki disease, particularly in children under the age of 4, and can be fatal in severe cases. Key features include persistent fever, hypotension, multiorgan involvement, and elevated inflammatory markers. International statistics indicate that the incidence of MIS-C among children with COVID-19 in the United States and other developed countries is less than 1%, classifying it as a relatively rare complication. However, the overall mortality rate is approximately 2%, which is significantly higher than the crude case fatality rate of 0.2% for COVID-19. Despite these statistics, many developing countries continue to face challenges in accurately identifying and diagnosing MIS-C.

Given the current landscape, organizations such as the World Health Organization (WHO) and the Centers for Disease Control and Prevention (CDC) have established treatment guidelines for MIS-C (1, 2). The therapeutic approach to MIS-C closely mirrors that of Kawasaki disease and encompasses symptomatic supportive care, immunomodulatory therapy, and antimicrobial treatment. In terms of immunomodulatory therapy, the majority of Children with MIS-C achieve superior disease control with IVIG and/or GCs. The CDC and the National Medical Products Administration (NMPA) advocate for IVIG and methylprednisolone as the treatments of choice for MIS-C (2, 3). The American College of Rheumatology currently endorses the use of IVIG and GCs, either as monotherapy or in combination (4). Spanish guidelines propose a staged approach to immunomodulatory therapy, with IVIG or GCs as preferred initial options, and recommend combining the two if there is no improvement or if the condition deteriorates (5). In the UK, a nationwide consensus management plan recommends IVIG as first-line therapy for Children with MIS-C, with GCs considered as a subsequent option if patients remain symptomatic, particularly with persistent fever, 24 h after IVIG administration (6). Initial treatment guidelines for MIS-C vary across countries, and no universally accepted preferred therapy exists. Recent evidence suggests that GCs alone may be a safe alternative to immunoglobulin or combination therapy. This finding is particularly significant given the cost and limited availability of IVIG in many countries, potentially offering new guidance for clinicians managing children with MIS-C.

This study was conducted to compare immunomodulatory therapies using IVIG, GCs, or a combination of IVIG and GCs, and to evaluate clinical outcomes including treatment failure, need for adjuvant immunotherapy, risk of left ventricular (LV) dysfunction, requirement for positive inotropic drug therapy, intensive care length of stay, fever duration, and duration of positive inotropic drug use. Through this systematic review and meta-analysis, we analyzed the best available evidence on the use and effectiveness of IVIG alone, GCs alone, and IVIG combined with GCs, identified superior treatment options, and provided more robust evidence.

## 2 Method

### 2.1 Search strategy and eligibility criteria

We conducted a meta-analysis adhering to the PRISMA (Preferred Reporting Items for Systematic Reviews and Meta-Analyses) guidelines. We systematically searched PubMed, Cochrane Library, EMBASE, Web of Science, and Medline for studies on GCs, IVIG, or combination therapy for MIS-C up to April 8, 2025. The detailed search strategy is provided in the online supplementary material (Supplementary Table S1). Articles identified through the systematic search were screened based on title, abstract, study design, and quality. Studies were included if they met all of the following criteria: (1) focused on IVIG, GCs, or combination therapy for MIS-C; (2) reported at least three outcomes of interest; (3) provided demographic information for Children with MIS-C. We excluded epidemiological studies, single-arm cohort studies, and studies lacking clinical data. Two authors (Junjie Lin and Yuan Yuan) independently reviewed and selected the articles, with disagreements resolved through team discussions.

### 2.2 Qualification criteria

#### 2.2.1 Inclusion criteria

Patients in each study were children clinically diagnosed with MIS-C.

MIS-C diagnosis was based on Centers for Disease Control and Prevention (CDC) and World Health Organization (WHO) guidelines (7, 8).

All observational or retrospective cohort studies published in English comparing the effects of IVIG, GCs, and IVIG plus GCs in the context of COVID-19.

#### 2.2.2 Exclusion criteria

Case reports, systematic reviews, meta-analyses, editorials, commentaries, reviews, studies with missing or insufficient data, and research published in languages other than English.

### 2.3 Data extraction

We developed an Excel spreadsheet template to systematically extract data across several key variables: author name, study year, number of participants, and study design. For each study, we categorized the treatment groups into IVIG, GCs, IVIG plus GCs, or no treatment, as defined in each study, and extracted the corresponding outcomes.

### 2.4 Outcomes and study population

#### 2.4.1 Primary outcomes

Need for inotropic support, treatment failure/persistent fever, left ventricular dysfunction, need for adjuvant immunotherapy, mortality, and coronary artery dilatation/aneurysm.

#### 2.4.2 Secondary outcomes

Intensive care unit (ICU) stay, duration of post-treatment fever, and duration of inotropic support.

#### 2.4.3 Study population

Children diagnosed with MIS-C.

### 2.5 Risk of bias assessment

The risk of bias for the included studies was evaluated using the Cochrane Non-Randomized Study Bias Risk Assessment Tool (ROBINS-I). Two researchers independently assessed bias risk across seven domains: (1) confounding bias, (2) selection bias, (3) intervention classification, (4) deviation from intended interventions, (5) missing data, (6) outcome measurement, and (7) selective reporting. Additionally, the Newcastle-Ottawa Scale (NOS) was employed to evaluate the quality of the included studies, with a focus on (1) cohort selection, (2) comparability, and (3) outcomes.

### 2.6 Statistical analysis

Study characteristics, baseline demographics, and outcome data were extracted into pre-specified data collection tables. For each selected trial, we recorded the median and interquartile range (IQR). When data followed a normal distribution, the median was used as a proxy for the mean, and the IQR was employed to estimate the standard deviation. Effect sizes were quantified using odds ratios (OR), standardized mean differences (SMD), and 95% confidence intervals (CI). Bayesian analysis was applied to generate overall ranking probabilities for each treatment, enabling each outcome to be ranked from most to least favorable. The rankings were visualized by calculating the surface under the cumulative ranking curve. All statistical analyses were performed using STATA 15.1, adhering to the intention-to-treat principle. This study was prospectively registered in PROSPERO (International Prospective Register of Systematic Reviews) under registration number CRD42023456156.

## 3 Result

### 3.1 Selection and characteristics of the studies

**Figure 1:**
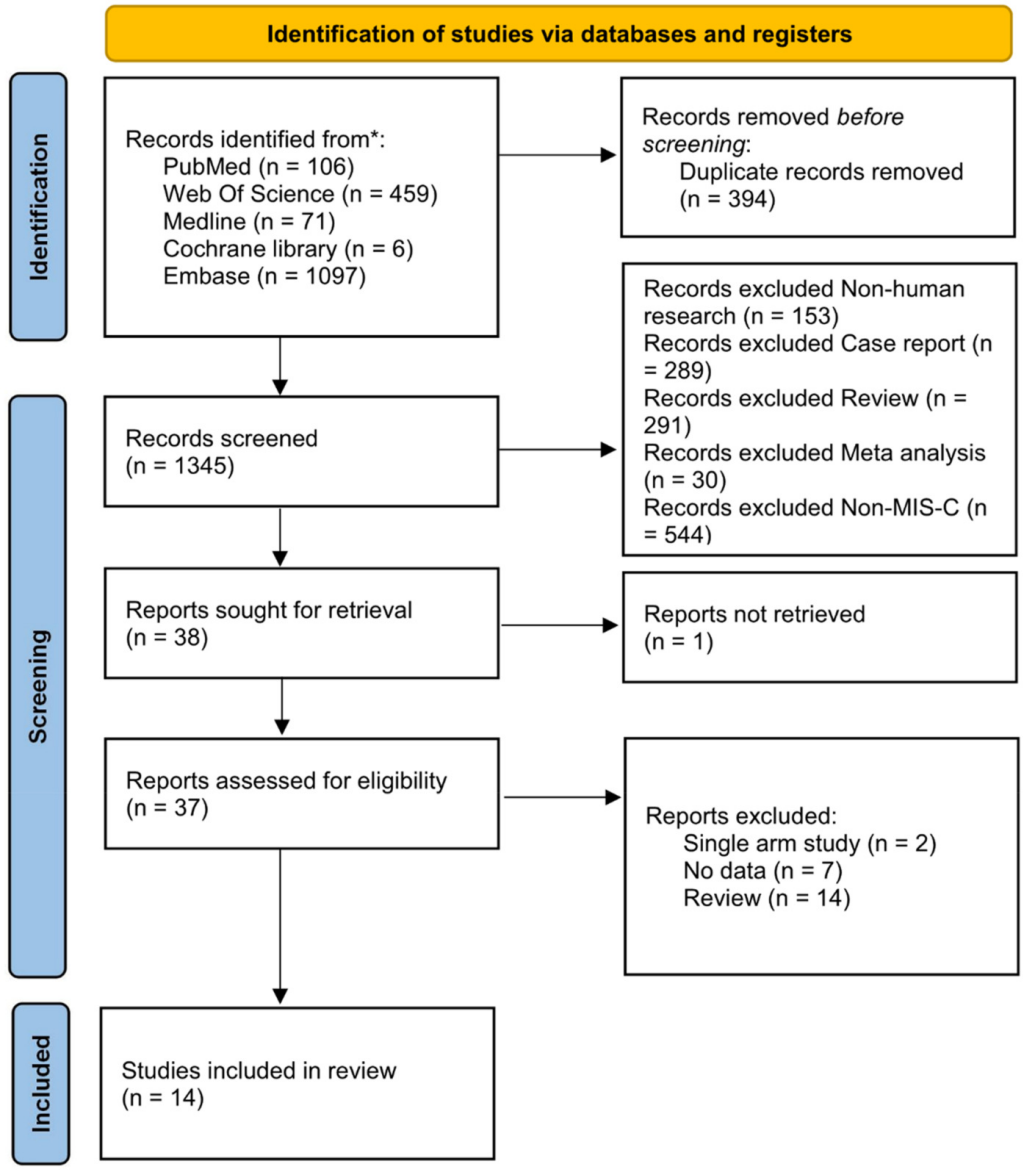
PRISMA flowchart showing selection of studies.

Our search identified a total of 1,739 trials from the central database and secondary searches. After removing 394 duplicates, 1,345 studies underwent preliminary screening. Titles and abstracts were evaluated against the inclusion criteria. Of these, 37 papers advanced to full-text review, and 14 studies were ultimately included in the quantitative analysis. The PRISMA flowchart outlines the study selection process (Figure 1), and the comparative network of primary and secondary outcomes is depicted in Supplementary Figure S1. The final analysis included 14 cohort studies involving 4,269 patients: 5 observational cohort studies (9–13), 8 retrospective cohort studies (14–21), and 1 conference abstract (22) (Table 1). To minimize bias, propensity score matching or inverse probability weighting was applied. All included studies were published post-2021.

**Table 1:** Baseline characteristics of the included studies.

Study	Year	No. of patients	Intervention	No. of patients	Age (year)	Male sex (%)	Outcomes
Bagri et al. (18)	2022	347	IVIG	28	4.25 (1.2–7)	64.3	The requirement of vasoactive/inotropic support on day 2 or beyond or need of mechanical ventilation on day 2 or beyond after initiation of immunomodulatory treatment or death during hospitalization in the treatment groups
			GC	82	6.7 (2.5–11)	54.9	
			IVIG + GC	237	7 (4–6.25)	64.5	
Harthan et al. (13)	2022	229	IVIG	33	5.5 (3.4–11)	57.6	The hospital and ICU LOS, hospital mortality, nosocomial bacterial infection, inotrope or ventilator requirement on or after hospital day 2, the number of days of inotropes, fever defervescence by day 3, and the day of normalization of inflammatory mediators
			GC	43	10 (5.3–15)	51.2	
			IVIG + GC	153	8.9 (5.5–12.0)	66	
Son et al. (20)	2021	330	IVIG	89	5.5 (2.5–10.5)	55	Cardiovascular dysfunction, LV dysfunction, inotrope use, adjunctive therapy, and persistence of fever
			IVIG + GC	241	8.6 (4.6–12.0)	56	
McArdle et al. (9)	2021	553	IVIG	246	7.0 (3.7–11.0)	64	Ventilated or death, improvement, treatment escalation, fever, death, any deterioration, LV dysfunction, and coronary artery dilation/aneurysm
			GC	99	8.8 (5.0–12.0)	60	
			IVIG + GC	208	8.8 (4.6–12.0)	61	
Tagarro et al. (14)	2022	132	IVIG	29	9.3 (5.9–12.6)	58.6	Discharge over time, the probability of switching to second-line treatment over time, and the persistence of fever 2 days after treatment
			GC	30	10.1 (7.1–8.5)	66.7	
			IVIG + GC	73	7.6 (4.1–8.4)	65.8	
Vukomanovic et al. (19)	2022	22	IVIG	10	13.4 ± 3.7	80	Treatment failure, LV dysfunction, ICU stays, laboratory parameters
			GC	12	11.8 ± 4.4	58.3	
Channon-Wells et al. (10)	2023	1,865	IVIG	680	6.8 (3.6–10.4)	61.2	Composite of inotropic or ventilator support from the second day after treatment initiation, or death, and time to improvement on an ordinal clinical severity scale, treatment escalation, clinical deterioration, fever, and coronary artery aneurysm occurrence and resolution
			GC	487	8.8 (5.1–12.1)	59.1	
			IVIG + GC	698	8.4 (4.5–11.3)	58.7	
Sugunan et al. (11)	2021	32	IVIG	6	3.5 (2.4–4.5)	–	Persistence of fever beyond 36 h after start of immunomodulation therapy, duration of ICU stay, mortality, need for repeat immunomodulation, time to normalization of CRP and persistence of coronary abnormalities at 2 weeks
			GC	26	8 (6–10.25)	–	
Devrim et al. (16)	2022	91	IVIG	42	–	–	The rate of hospitalization in the PICU, duration of fever, and length of stay in the hospital
			IVIG + GC	49	–	–	
Gowin et al. (12)	2022	167	IVIG	76	8.7 (4.6–11.3)	61	Persistent or recurrent fevers, need for adjunctive immunomodulatory therapy or hemodynamic support
			IVIG + GC	91	9.4 (5.2–12.5)	71	
Villacis-Nunez et al. (15)	2022	179	GC	111	–	–	Failure of initial therapy, presence of complications, cardiovascular outcomes, fever duration, length of hospital and ICU stays, corticosteroid use duration, and need for readmission
			IVIG + GC	68	–	–	
Ouldali et al. (17)	2021	106	IVIG	72	8.7 (4.6–12.0)	48	Treatment failure, adjunctive therapy, inotropic support, LV dysfunction, and duration of PICU stay
			IVIG + GC	34	9.1 (4.7–13.1)	53	
Villacis Nunez et al. (22)	2021	216	IVIG	31	7 (5–9.5)	71	Failure of initial therapy, duration of fever and vasoactive support, Coronary abnormalities, length of ICU stay, hospital length of stay, readmission, and number of emergency room (ER) visits up to 6 months after discharge
			GC	69	10 (6–14)	60.9	
			IVIG + GC	116	8 (5–12)	62.1	
Phan et al. (21)	2025		GC	316	7.125 (4–9.5)	66.5	Treatment failure, Duration of fever (days), Duration of inotropic support (days), Requiring respiratory or inotropic support (%) within three initial days, Hospital length of stay (LOS) (days) and hospital LOS ≥7 days (%), PICU LOS (days) and PICU LOS ≥3 days (%), Reduced left ventricular (LV) ejection fraction (EF) <55% (%), Coronary artery dilation or aneurysm (%)

### 3.2 Adjuvant immunotherapy

**Figure 2:**
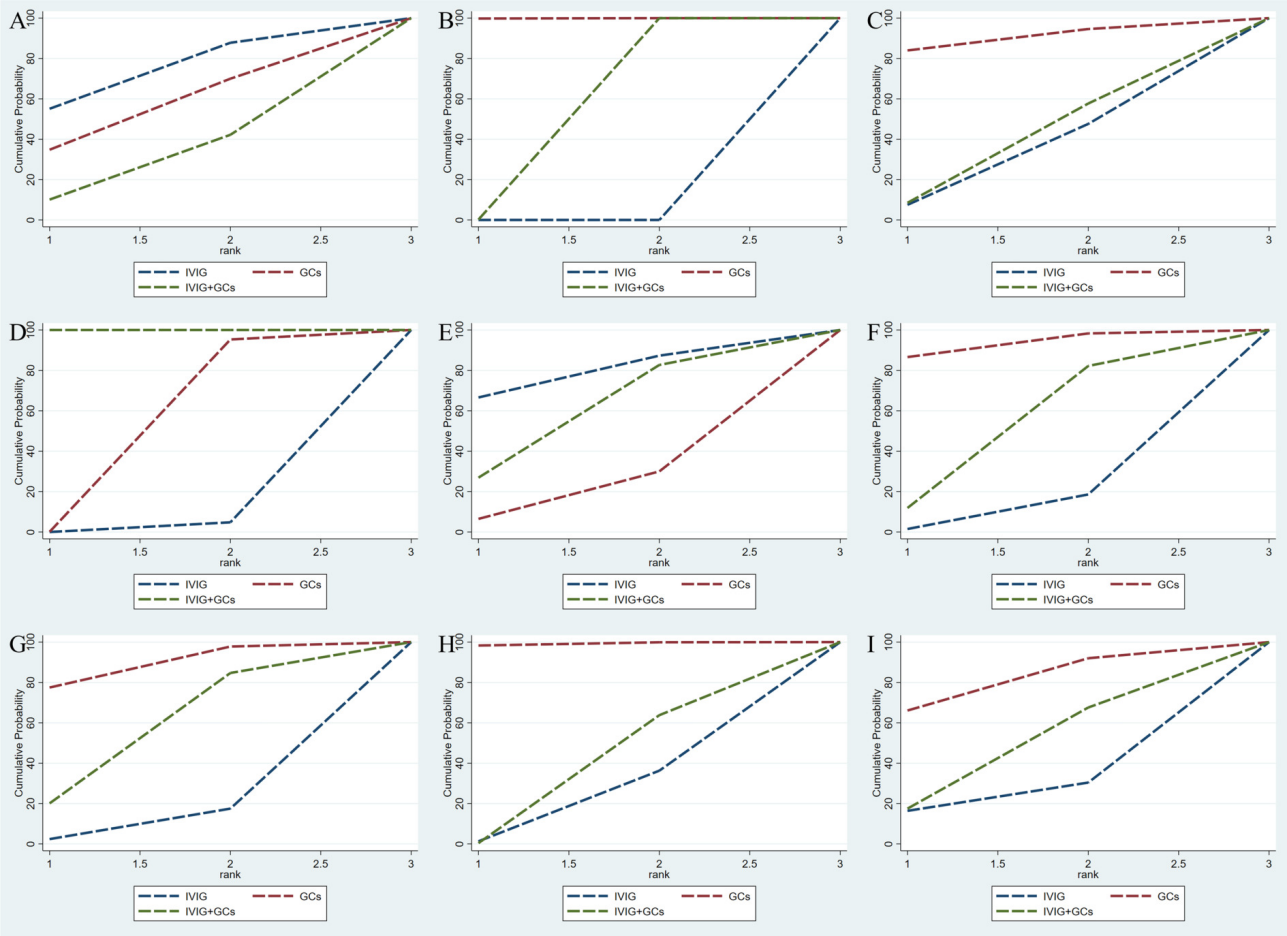
Cumulative probability indicates the ranking of efficacy on the following: adjuvant immunotherapy **(A)**, persistent fever/failure of treatment **(B)**, need inotropic support **(C)**, left heart dysfunction **(D)**, mortality **(E)**, coronary artery dilatation/aneurysm **(F)**, ICU long of stay **(G)**, duration of fever **(H)**, duration of inotropic support **(I)**. The larger the surface under the curve, the better the rank of the intervention being the stipulation.

**Figure 3:**
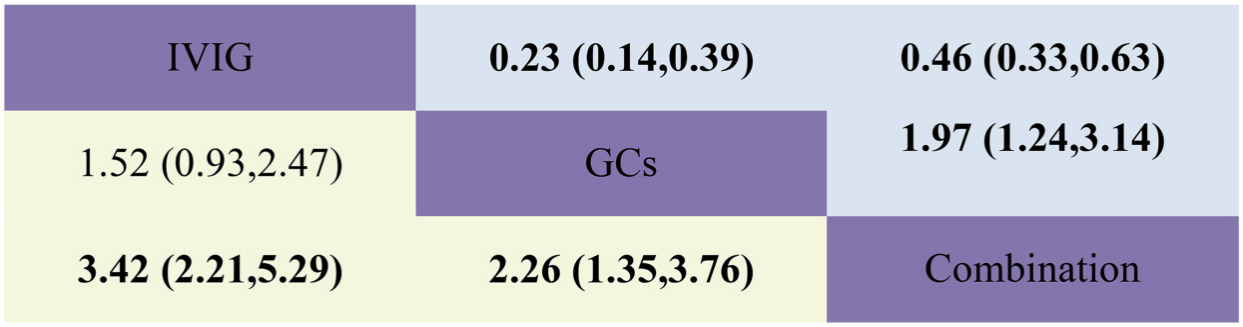
Head-to-head comparisons of interventions for persistent fever/failure (upper shaded boxes) and adjuvant immunotherapy (lower shaded boxes). Data are odds ratios with 95% credible intervals. The figure should be read from left to right: for persistent fever/failure to treat comparisons (upper shaded boxes), odds ratios <1 favour the column defining the treatment, whereas for persistent fever/failure to treat comparisons (lower shaded boxes), odds ratios >1 favour the row defining the treatment.

**Figure 4:**
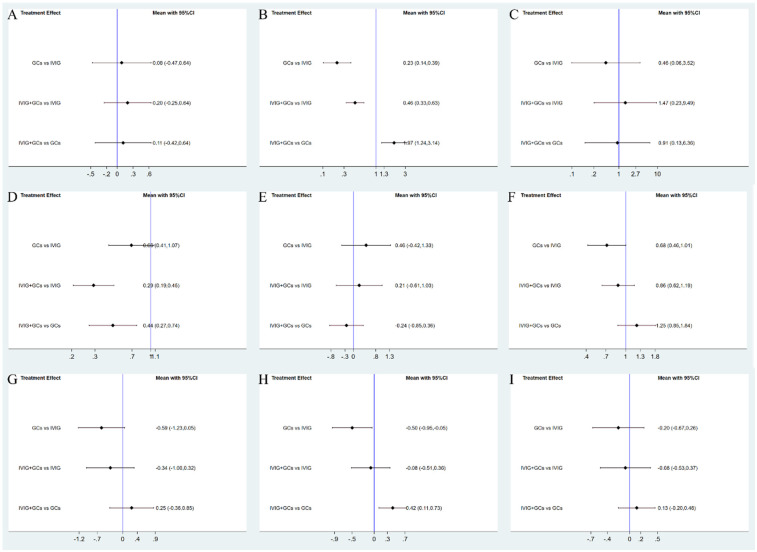
Forest plots. Shown are forest plots from the network meta-analysis of all trials for Adjuvant immunotherapy **(A)**, persistent fever/failure of treatment **(B)**, need inotropic support **(C)**, left heart dysfunction **(D)**, mortality **(E)**, coronary artery dilatation/aneurysm **(F)**, ICU long of stay **(G)**, duration of fever **(H)**, duration of inotropic support **(I)**.

Adjuvant immunotherapy was defined as fever persisting beyond 36 h post-initial immunomodulatory therapy or clinical deterioration post-treatment. In such cases, recurrent immunoregulation was considered, irrespective of the time elapsed since the completion of the initial treatment regimen. Our aggregated data demonstrates that, among the 1,225 patients in the IVIG group, 721 needed additional adjuvant immunotherapy; of the 663 patients in the GC group, 314 required it; and within the IVIG + GC group of 1,246 patients, 352 needed additional adjuvant immunotherapy. The aggregated results demonstrated that the proportion of patients necessitating additional adjuvant immunotherapy was significantly lower in the IVIG + GCs group compared to the IVIG group [OR 0.29, 95% CI (0.19, 0.45)] and in the GCs group relative to the IVIG group [OR 0.66, 95% CI (0.41, 1.07)]. Moreover, the IVIG + GCs group exhibited a significantly lower proportion than the GCs group [OR 0.44, 95% CI (0.27, 0.74)] (Figure 2A; Supplementary Figure S2A; Figure 3). The SUCRA ranking corroborated these findings, indicating that the IVIG + GCs group had the lowest proportion of patients requiring additional adjuvant immunotherapy, followed by the GCs group. Conversely, the majority of patients in the IVIG group still required adjuvant immunotherapy post-treatment (Figure 4A; Supplementary Figure S3A).

### 3.3 Persistent fever/treatment failure

Persistent fever or treatment failure is defined as a body temperature above 38.0°C on Day 2 or later after immunotherapy. Our aggregated data indicate the following: among 331 patients in the IVIG group, 145 required additional treatment for failure; among 188 patients in the GCs group, 31 required additional treatment for failure; and among 452 patients in the IVIG plus GCs group, 129 required additional treatment for failure. The combined results show that the proportion of treatment failure was significantly lower in the IVIG plus GCs group than in the IVIG group [OR 0.46, 95% CI (0.33, 0.63)], in the GCs group than in the IVIG plus GCs group [OR 0.51, 95% CI (0.32, 0.81)], and in the GCs group than in the IVIG group [OR 0.23, 95% CI (0.14, 0.39)] (Figure 2B; Supplementary Figure S2B; Figure 3). The SUCRA ranking indicates that the proportion of treatment failure was lowest in the GCs group, followed by the IVIG plus GCs group, and then the IVIG group (Figure 4B; Supplementary Figure S3B).

### 3.4 Need for inotropic support

**Figure 5:**
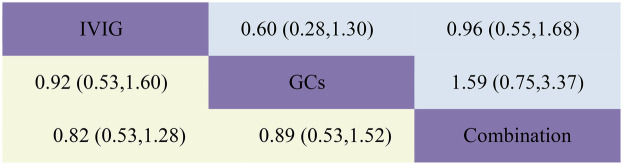
Head-to-head comparisons of interventions for LVEF (upper shaded panels) and need for inotropic support (lower shaded panels). Data are odds ratios with 95% credible intervals. The figure should be read from left to right: for comparisons of LVEF (upper shaded fields), odds ratios <1 favour the column defining the treatment, whereas for need for inotropic support (lower shaded fields), odds ratios >1 favour the row defining the treatment.

The requirement for inotropes was defined as the use of any scheduled inotropic or vasopressor support on Day 2 following immunotherapy. Our aggregated data reveal that among the 438 patients in the IVIG group, 85 required additional inotropes; among the 154 patients in the GCs group, 38 required additional inotropes; and among the 393 patients in the IVIG plus GCs group, 78 required additional inotropes. The combined results indicated that the proportion of patients requiring additional inotropes was lower in the IVIG group than in the IVIG plus GCs group (OR 0.82, 95% CI 0.53–1.28), lower in the GCs group than in the IVIG plus GCs group (OR 0.89, 95% CI 0.53–1.52), and slightly lower in the IVIG group than in the GCs group (OR 0.92, 95% CI 0.53–1.60) (Figure 2C; Supplementary Figure S2C; Figure 5). The SUCRA ranking further demonstrated that the IVIG group had the lowest need for inotropes, followed by the GCs group, and then the IVIG plus GCs group (Figure 4C; Supplementary Figure S3C).

### 3.5 Left heart dysfunction

The study defined left ventricular dysfunction as a left ventricular ejection fraction (LVEF) of less than 55% on echocardiography. In our aggregated data reveal that, among the 433 patients in the IVIG group, 59 developed left ventricular dysfunction; among the 185 patients in the GCs group, 22 developed left ventricular dysfunction; and among the 440 patients in the IVIG plus GCs group, 50 developed left ventricular dysfunction. The combined results showed that the proportion of patients developing left ventricular dysfunction was lower in the IVIG plus GCs group than in the IVIG group [OR 0.96, 95% CI (0.55, 1.68)], lower in the GCs group than in the IVIG plus GCs group [OR 0.63, 95% CI (0.30, 1.33)], and lower in the GCs group than in the IVIG group [OR 0.60, 95% CI (0.28, 1.30)] (Figure 2D; Supplementary Figure S2D; Figure 5). The SUCRA ranking indicated that the GCs group had the lowest proportion of patients developing left ventricular dysfunction, followed by the IVIG plus GCs group, and then the IVIG group (Figure 4D; Supplementary Figure S3D).

### 3.6 Mortality

**Figure 6:**
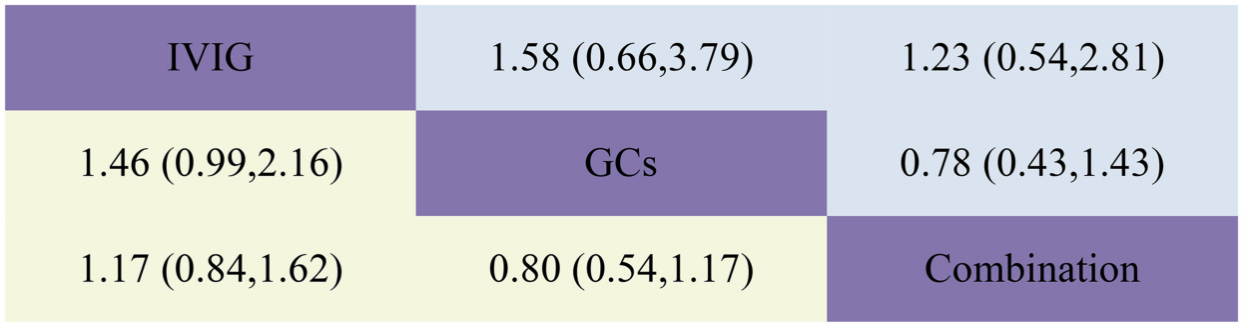
Head-to-head comparisons of interventions for mortality (upper shaded fields) and coronary artery dilatation/aneurysm (lower shaded fields). The figure should be read from left to right: for comparisons of Mortality (upper shaded fields), odds ratios <1 favour the column defining the treatment, whereas for Coronary Artery Dilatation (lower shaded fields), odds ratios >1 favour the row defining the treatment.

Post-treatment mortality rates are a critical focus of our analysis, our aggregated data reveal that, in the IVIG group, 8 patients died out of 712; in the GCs group, 17 patients died out of 494; and in the IVIG plus GC group, 36 patients died out of 1,232. Our statistical analysis indicates that, compared to the GCs group (OR 0.63, 95% CI 0.26, 1.52) and the GCs combined with IVIG group [OR 0.81, 95% CI (0.36, 1.84)], the use of IVIG alone is associated with the lowest mortality rate (Figure 2E; Supplementary Figure S2E; Figure 6). However, this difference was not statistically significant. According to the SUCRA ranking, IVIG emerged as the most favorable option for reducing mortality among the three treatment regimens (Figure 4E; Supplementary Figure S3E).

### 3.7 Coronary artery dilatation/aneurysm

Our aggregated data show that in the IVIG group, 87 of 722 patients had persistent coronary artery dilation/aneurysms after treatment; in the GCs group, 48 of 510 patients had these conditions; and in the IVIG plus GC group, 78 of 697 patients presented with coronary artery dilation/aneurysms. Compared with IVIG monotherapy [OR 0.68, 95% CI (0.46, 1.01)] and IVIG combined with GCs therapy [OR 0.80, 95% CI (0.54, 1.17)], GCs monotherapy is linked to a lower incidence of coronary artery dilation in patients (Figure 2F; Supplementary Figure S2F; Figure 6). According to the SUCRA ranking, GCs monotherapy results in the lowest incidence of coronary artery dilation/aneurysm, followed by IVIG and GCs, while the combination of IVIG monotherapy underperforms in comparison (Figure 4F; Supplementary Figure S3F).

### 3.8 ICU length of stay

**Figure 7:**
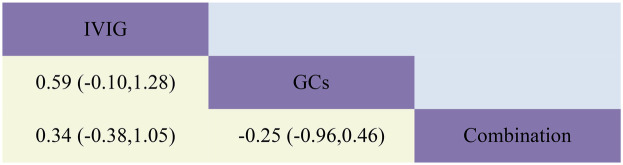
Head-to-head comparisons of interventions for ICU length of stay (lower shaded fields). The figure should be read from left to right: for comparisons of ICU length of stay (lower shaded fields), SMD >0 favour the line defining the treatment.

The combined results demonstrated that compared to IVIG monotherapy, IVIG combined with GCs treatment was associated with a relatively shorter ICU length of stay [SMD −0.34, 95% CI (−1.00, 0.32)]. Furthermore, the GCs group exhibited a shorter ICU length of stay compared to both the IVIG plus GCs group [SMD −0.25, 95% CI (−0.85, 0.36)] and the IVIG group [SMD −0.59, 95% CI (−1.23, 0.05)] (Figure 2G; Supplementary Figure S2G; Figure 7). The SUCRA ranking indicated that the GCs group had the shortest ICU length of stay, followed by the IVIG plus GCs group, while the IVIG group showed the least reduction in ICU length of stay (Figure 4G; Supplementary Figure S3G).

### 3.9 Duration of fever

**Figure 8:**
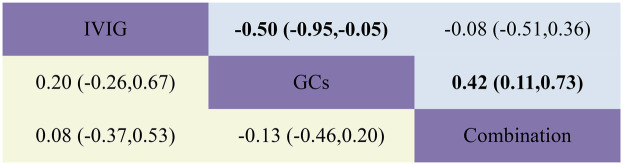
Head-to-head comparisons of interventions for duration of fever (upper shaded fields) and duration of inotropic support (lower shaded panels). The figure should be read from left to right: for comparisons of duration of inotropic support (lower shaded fields), SMD >0 favour the line defining the treatment.

The combined results revealed that, compared with the IVIG plus GCs group, patients in the GCs monotherapy group exhibited a reduced duration of fever [SMD −0.42, 95% CI (−0.73, −0.11)]. The IVIG plus GCs group demonstrated a shorter fever duration than the IVIG group [SMD −0.08, 95% CI (−0.51, 0.36)], and the GC group showed a significantly shorter fever duration than the IVIG group [SMD −0.50, 95% CI (−0.95, −0.05)] (Figure 2H; Supplementary Figure S2H; Figure 8). The SUCRA ranking demonstrated that the GCs group was the most effective in reducing fever duration, followed by the IVIG plus GCs group, with the IVIG group being the least effective (Figure 2H; Supplementary Figure S2H).

### 3.10 Duration of inotropic support

The combined analysis revealed that the duration of inotropic support was reduced in the IVIG plus GCs group compared to the IVIG group [SMD −0.08, 95% CI (−0.53, 0.37)], further shortened in the GCs group relative to the IVIG plus GCs group [SMD −0.13, 95% CI (−0.46, 0.20)], and most notably reduced in the GCs group when contrasted with the IVIG group [SMD −0.20, 95% CI (−0.67, 0.26)] (Figure 2I; Supplementary Figure S2I; Figure 8). According to the SUCRA ranking, the GCs group demonstrated the most substantial reduction in the duration of inotropic support, followed by the IVIG plus GCs group, with the IVIG group exhibiting the least effect (Figure 2I; Supplementary Figure S2I).

### 3.11 Risk of bias and quality assessment

**Figure 9:**
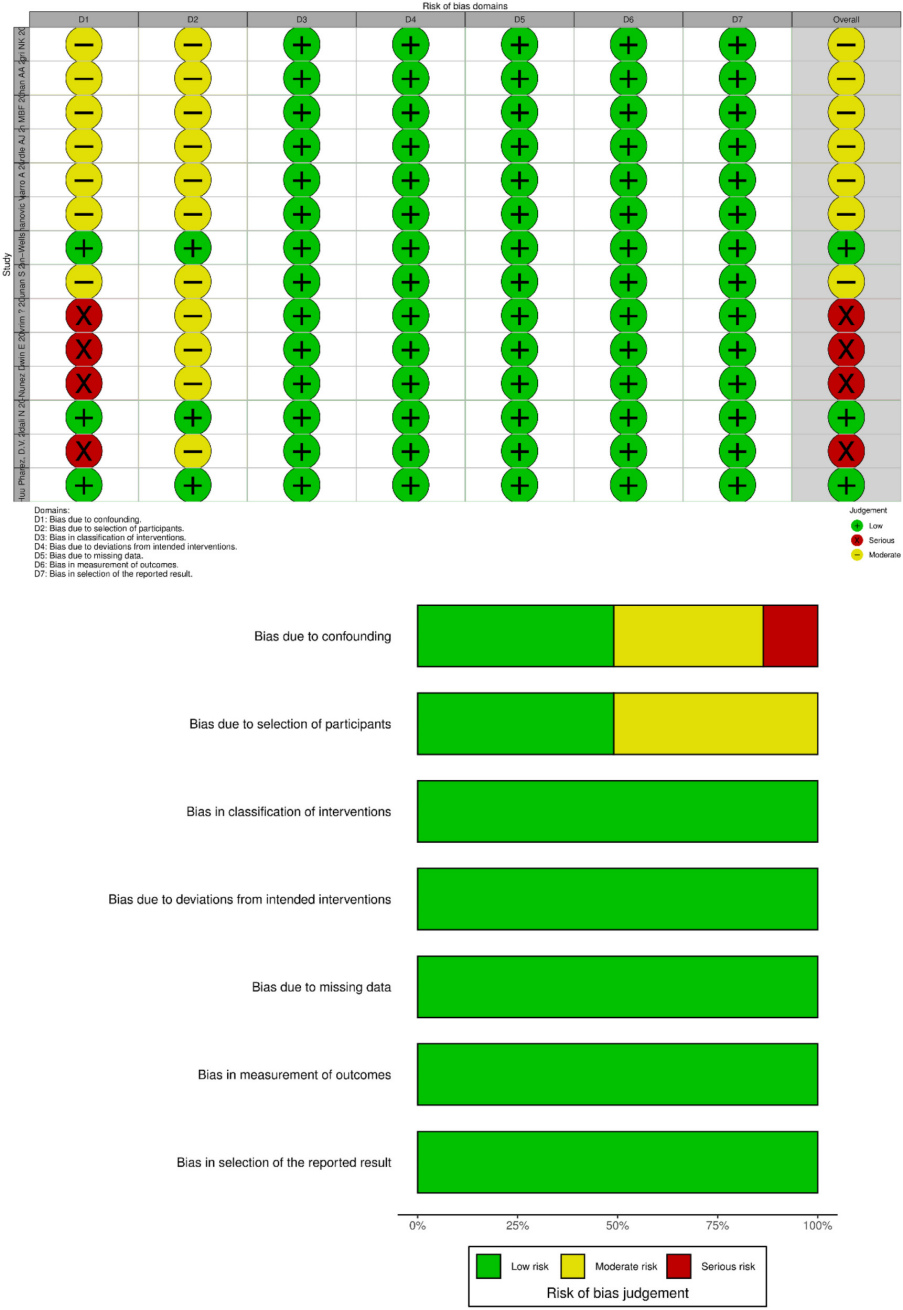
Risk of bias assessment.

Given that all included studies were non-randomized controlled trials, the Cochrane Non-Randomized Intervention Trials Bias Risk Assessment Tool (ROBINS-I) was employed to evaluate the risk of bias in the included studies. Reviewer pairs assessed seven domains related to bias at both the study and outcome levels. Each domain was classified as having a “high risk of bias,” “moderate risk of bias,” or “low risk of bias.” Four studies, including those by Villacis-Nunez et al. (2022) (15), Devrim et al. (16), and Villacis Nunez et al. (2021) (22), did not adequately control for confounding factors and were consequently rated as having a high risk of bias in this domain. In contrast, seven studies, such as those by Harthan et al. (13), Bagri et al. (18), and Phan et al. (21), implemented appropriate controls for confounding factors and were rated as having a moderate risk of bias. A moderate risk of bias was also noted regarding participant selection and reported results (Figure 9).

The Newcastle-Ottawa Scale (NOS) was utilized to assess the quality of the included studies. The NOS scores range from 1 to 10, with scores of 1–3 indicating low quality, 4–6 indicating moderate quality, and 7–10 indicating high quality. Funnel plots for each outcome showed no evidence of publication bias (Supplementary Figure S3), and consistency tests confirmed no significant inconsistencies across all outcomes.

## 4 Discussion

MIS-C, one of the most severe complications following SARS-CoV-2 infection in children, is marked by a systemic hyperinflammatory state. It can rapidly progress to shock, cardiopulmonary failure, and even death within 3–4 weeks of acute infection (23), particularly in low- and middle-income countries (22). However, there is substantial debate regarding first-line immunomodulatory treatments for MIS-C. The 2022 American College of Rheumatology guidelines endorse IVIG as first-line therapy. In contrast, the 2023 WHO guidelines (8) and the 2021 NIH guidelines (24) advocate against IVIG monotherapy as a first-line intervention unless GCs are contraindicated. The 2022 EAACI guidelines propose that corticosteroid monotherapy may be considered as a first-line immunomodulatory approach (25). These discrepancies in international guidelines present a significant challenge for clinicians in selecting appropriate treatment strategies. Furthermore, the limited availability of IVIG in many countries complicates treatment decisions. Additionally, the question of whether IVIG monotherapy or IVIG combined with GCs is more beneficial than GCs alone remains unresolved and requires robust evidence to determine which treatment should be prioritized.

After conducting a systematic search and stringent screening process, this study included 14 studies, comprising 13 cohort studies and 1 conference abstract, encompassing 4,269 Children with MIS-C. Given the clinical similarities between MIS-C and Kawasaki disease, and the established role of IVIG in Kawasaki disease treatment, IVIG has naturally become a key focus of research. Moreover, GCs have accumulated extensive clinical evidence in the management of inflammatory diseases, providing a rationale for their use in MIS-C treatment. Among the 14 included studies, 13 evaluated the efficacy of IVIG, 10 focused on GCs, and 12 explored the effects of IVIG combined with GCs. This systematic review and meta-analysis strictly adhered to the PRISMA 2020 guidelines and was registered on the PROSPERO platform. The efficacy results derived from pairwise and network meta-analyses demonstrated that GCs alone were significantly superior to IVIG alone and IVIG combined with GCs in reducing fever duration and treatment failure rates. Compared to IVIG alone and GCs alone, IVIG combined with GCs showed significant benefits in reducing the need for additional immunotherapy. Furthermore, GCs are also beneficial in reducing coronary artery dilation/aneurysms, shortening ICU length of stay, decreasing the duration of inotropic support, and lowering the post-treatment incidence of residual left ventricular dysfunction. Overall, GCs demonstrate favorable therapeutic effects and have a positive impact on various clinical outcomes.

Preliminary mechanistic studies indicate that the primary pathophysiological mechanism of MIS-C involves the uncontrolled activation of inflammatory cascades in response to SARS-CoV-2 infection (26, 27). GCs exert their effects by suppressing pro-inflammatory gene promoter activity through genomic mechanisms while enhancing the expression of anti-inflammatory mediators. Additionally, the trans-repression effects of GCs inhibit the expression of immunomodulatory and pro-inflammatory proteins, such as cytokines (IL-1, IL-2, IL-6, TNF-α, IFN-γ) and prostaglandins. The rapid non-genomic effects of GCs also play a critical role; high-dose administration can rapidly attenuate inflammation by inhibiting the expression of cytokines (e.g., TNF-α, IL-6, IL-1α, IL-1β) and chemokines (e.g., CXCL9 and CXCL10) within minutes, thereby suppressing vasodilation and vascular permeability. IVIG reduces IL-6 levels on days 3 and 4 of illness onset, with CXCL9 and CXCL10 levels decreasing only on day 5 (19, 28, 29). Based on these mechanisms, we posit that GCs may benefit MIS-C by systemically suppressing SARS-CoV-2-induced inflammatory responses (34).

Coronary artery ectasia or aneurysms represent one of the more frequent complications encountered in children with MIS-C, and they may significantly compromise long-term cardiovascular health. IVIG is employed in treating MIS-C due to the clinical parallels between MIS-C and Kawasaki disease; IVIG is a well-established therapy for mitigating the risk of coronary artery aneurysms (30). However, there are concerns that initiating treatment solely with GCs might be linked to a heightened risk of coronary artery aneurysms. Research conducted by Phuc Huu Phan and colleagues revealed no statistically significant disparity in the incidence of coronary artery ectasia or aneurysms when comparing the group treated exclusively with GCs to the group that received a combination of GCs and IVIG (19.5% vs. 14.61%, respectively, *p* = 0.557) (21). Similarly, the study by Samuel Channon-Wells and colleagues demonstrated that the incidence rate of coronary artery aneurysms in patients who initially received glucocorticoids was comparable to that observed in patients treated with IVIG, whether as monotherapy or in conjunction with GCs (12). The findings of this study suggest that initiating treatment with GCs alone is not associated with an increased long-term risk of coronary artery ectasia or aneurysms in children with MIS-C.

Although most Children with MIS-C recover with timely treatment, the disease can still be fatal. Studies indicate that severe cardiovascular involvement is associated with MIS-C mortality (31). Data from the U.S. CDC show higher PICU admission rates and lower mortality (0.8%) in high-income countries (32), with an overall MIS-C mortality rate of approximately 1.7% (33). This may be attributed to limited medical resources, delayed diagnosis, and pre-existing conditions in low- and middle-income countries. Based on our meta-analysis, no significant differences were observed in MIS-C mortality among the three treatment regimens. Therefore, we recommend conducting high-quality randomized controlled trials to further confirm the efficacy of GCs in MIS-C treatment. For patients unresponsive or minimally responsive to initial treatment, additional adjunctive immunotherapies, such as infliximab and anakinra, may be necessary. Network meta-analysis results suggest that GCs combined with IVIG can reduce the need for additional immunotherapy.

Our meta-analysis has several strengths: it focuses on comparing first-line treatment regimens for MIS-C, which holds clinical significance, particularly given the notable discrepancies in global MIS-C treatment guidelines. This study is the first to employ network meta-analysis to systematically compare the efficacy of IVIG, GCs, and IVIG combined with GCs, providing comprehensive evidence-based medical evidence for clinicians. For countries with limited IVIG resources, the study explores the feasibility of GCs as first-line therapy, offering important practical value. Additionally, the study covers multiple databases with searches updated to April 2025, ensuring the timeliness and comprehensiveness of the data.

However, some limitations exist in this meta-analysis. First, while the study focuses on MIS-C treatment, it does not compare other potential treatment options (e.g., biologics), which may limit the comprehensiveness of the conclusions. Second, the included studies are based on observational data, lacking high-quality evidence from RCTs, which may affect causal inferences. Furthermore, some included studies did not adequately control for confounding factors (e.g., disease severity, treatment dosage), potentially introducing bias. Lastly, due to the absence of RCT data, the evidence level of the results is low, which may not fully support the development of clinical guidelines. Additionally, the results are primarily based on data from high-income countries, and their applicability in low- and middle-income countries requires further validation.

Our study demonstrates that GC monotherapy significantly reduces treatment failure rates and persistent fever duration, while combination therapy significantly reduces the need for adjunctive immunotherapy. For countries with limited access to IVIG, initiating GCs as first-line therapy may be a viable option.

## Data Availability

The original contributions presented in the study are included in the article/Supplementary Material, further inquiries can be directed to the corresponding author.
