# Supplementary material for: Glucocorticoids and immunoglobulin alone or in combination in the treatment of multisystemic inflammatory syndrome in children: a systematic review and network meta-analysis
Source: Front Pediatr. 2025 May 29;13:1545788. doi: 10.3389/fped.2025.1545788 (PMC12158936; doi:10.3389/fped.2025.1545788)
Supplement: Supplementary file 1 [file Datasheet1.docx]

**Glucocorticoids and immunoglobulin alone or in combination in the treatment of multisystemic inflammatory syndrome in children: a systematic review and network meta-analysis**

**SUPPLEMENTARY APPENDIX**

**Supplementary Table 1.Quality of Evidence Assessment**

**Supplementary Table 2.Risk of Bias**

**Supplementary Figure 1.Network graphs**

**Supplementary Figure 2.SUCRA**

**Supplementary Figure 3.Funnel plot.**

**Supplementary Figure4.Conformity Test.**

sTable 1.Quality of Evidence Assessment

| Study | Selection | | | | Comparability | Outcome | | | Scores |
| --- | --- | --- | --- | --- | --- | --- | --- | --- | --- |
|  | Representativeness of the Exposed Cohort | Selection of the Non-Exposed Cohort | Ascertainment of Exposure | Demonstration That Outcome of Interest Was Not Present at Start of Study | Comparability of Cohorts on the Basis of the Design or Analysis | Assessment of Outcome | Was Follow-Up Long Enough for Outcomes to Occur | Adequacy of Follow Up of Cohorts |  |
| Bagri NK 2022 | ☆ | - | ☆ | - | ☆☆ | ☆ | - | ☆ | 6 |
| Harthan AA 2022 | ☆ | - | ☆ | - | ☆☆ | ☆ | - | ☆ | 6 |
| Son MBF 2021 | ☆ | - | ☆ | - | ☆☆ | ☆ | - | ☆ | 6 |
| McArdle AJ 2021 | ☆ | - | ☆ | - | ☆☆ | ☆ | - | ☆ | 6 |
| Tagarro A 2022 | ☆ | - | ☆ | - | ☆☆ | ☆ | - | ☆ | 6 |
| Vukomanovic V 2022 | ☆ | - | ☆ | - | ☆ | ☆ | - | ☆ | 5 |
| Channon-Wells S 2023 | ☆ | - | ☆ | - | ☆☆ | ☆ | - | ☆ | 6 |
| Sugunan S 2021 | ☆ | - | ☆ | - | ☆ | ☆ | - | ☆ | 5 |
| Devrim İ 2022 | ☆ | - | ☆ | - | ☆ | ☆ | - | ☆ | 5 |
| Gowin E 2022 | ☆ | - | ☆ | - | ☆ | ☆ | - | ☆ | 5 |
| Villacis-Nunez DS 2022 | ☆ | - | ☆ | - | ☆☆ | ☆ | - | ☆ | 6 |
| Ouldali N 2021 | ☆ | - | ☆ | - | ☆☆ | ☆ | - | ☆ | 6 |
| Nunez, D.V. 2021 | ☆ | - | ☆ | - | ☆ | ☆ | - | ☆ | 5 |
| Phuc Huu Phan 2025 | ☆ | - | ☆ | - | ☆☆ | ☆ | - | ☆ | 6 |

PS: NOS scale full score of 10 points, 1-3 is classified as low quality, 4-6 is classified as medium quality, 7-10 is classified as high quality

sTable 2.Risk of Bias

| Study | Confounding  (all  outcomes) | Selection of  Participants | Classification  of the  Intervention | Deviation  From  Intended  Interventions  (assignment) | Missing Data  (all  outcomes) | Measurement  of Outcomes  (all  outcomes) | Selection of  Reported  Results (all  outcomes) | Overall |
| --- | --- | --- | --- | --- | --- | --- | --- | --- |
| Bagri NK 2022 | Moderate | Moderate | Low | Low | Low | Low | Low | Moderate |
| Harthan AA 2022 | Moderate | Moderate | Low | Low | Low | Low | Low | Moderate |
| Son MBF 2021 | Moderate | Moderate | Low | Low | Low | Low | Low | Moderate |
| McArdle AJ 2021 | Moderate | Moderate | Low | Low | Low | Low | Low | Moderate |
| Tagarro A 2022 | Moderate | Moderate | Low | Low | Low | Low | Low | Moderate |
| Vukomanovic V 2022 | Moderate | Moderate | Low | Low | Low | Low | Low | Moderate |
| Channon-Wells S 2023 | Low | Low | Low | Low | Low | Low | Low | Low |
| Sugunan S 2021 | Moderate | Moderate | Low | Low | Low | Low | Low | Moderate |
| Devrim İ 2022 | Serious | Moderate | Low | Low | Low | Low | Low | Serious |
| Gowin E 2022 | Serious | Moderate | Low | Low | Low | Low | Low | Serious |
| Villacis-Nunez DS 2022 | Serious | Moderate | Low | Low | Low | Low | Low | Serious |
| Ouldali N 2021 | Low | Low | Low | Low | Low | Low | Low | Low |
| Nunez, D.V. 2021 | Serious | Moderate | Low | Low | Low | Low | Low | Serious |
| Phuc Huu Phan 2025 | Low | Low | Low | Low | Low | Low | Low | Low |

PS:Risk of Bias Assessment Following the Cochrane Risk-of-bias Tools for Nonrandomized Studies of Interventions (ROBINS-I)22,Following the ROBINS-I tool, the categories for risk of bias judgements are “Low risk,” “Moderate risk,” “Serious risk,” and “Critical risk” of bias.

sFigure1.Network graphs


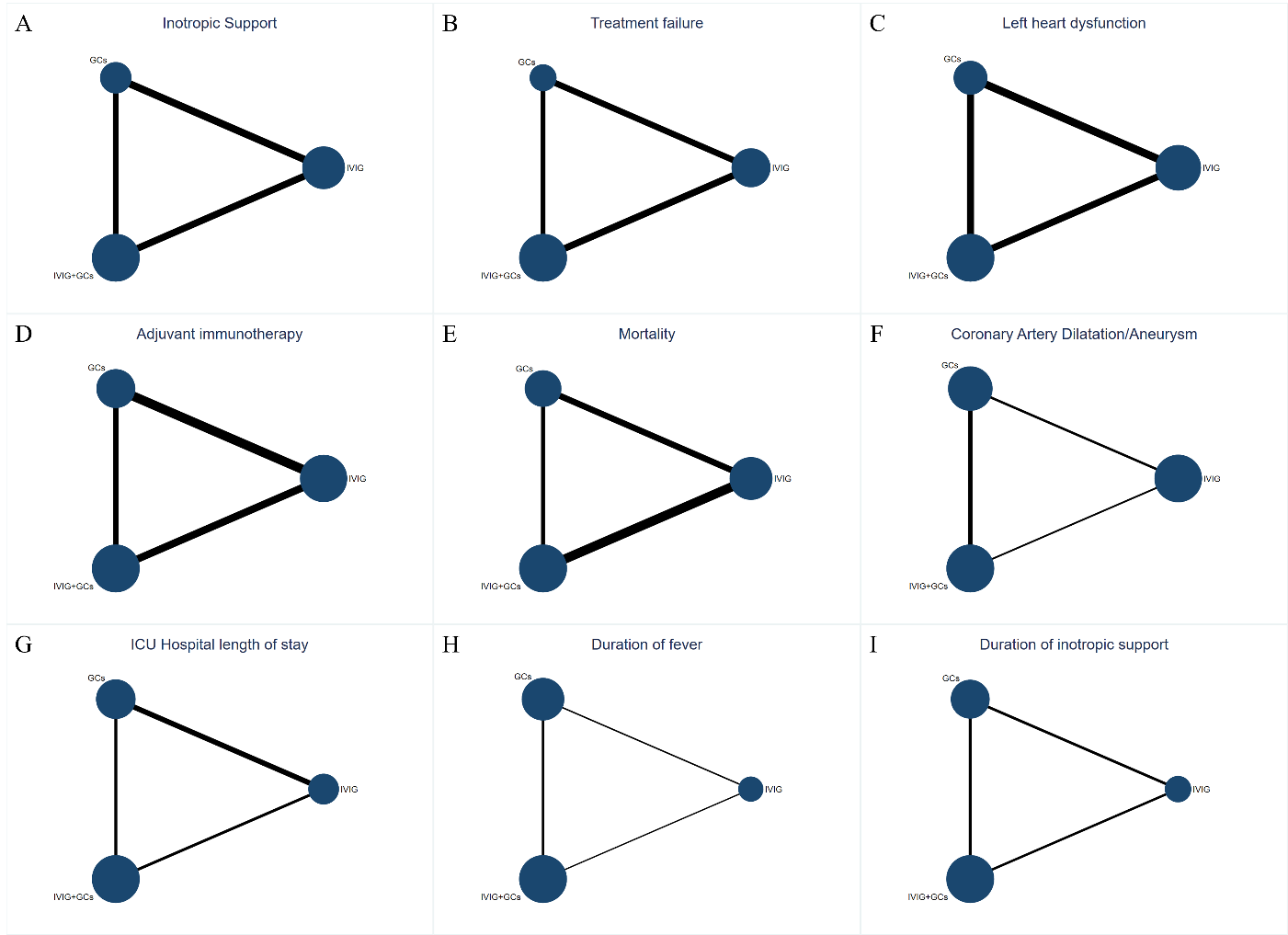


Network graphs. Network of eligible comparisons for Adjuvant immunotherapy (A) , Persistent fever / failure of treatment (B), Need inotropic support (C), Left heart dysfunction (D), Mortality (E), Coronary Artery Dilatation (F), ICU long of stay (G), Duration of fever (H),Duration of inotropic support (I). The width of lines is proportional to the number of studies comparing every pair of interventions. The size of nodes is proportional to the number of participants assigned to receive the intervention.

sFigure2.SUCRA


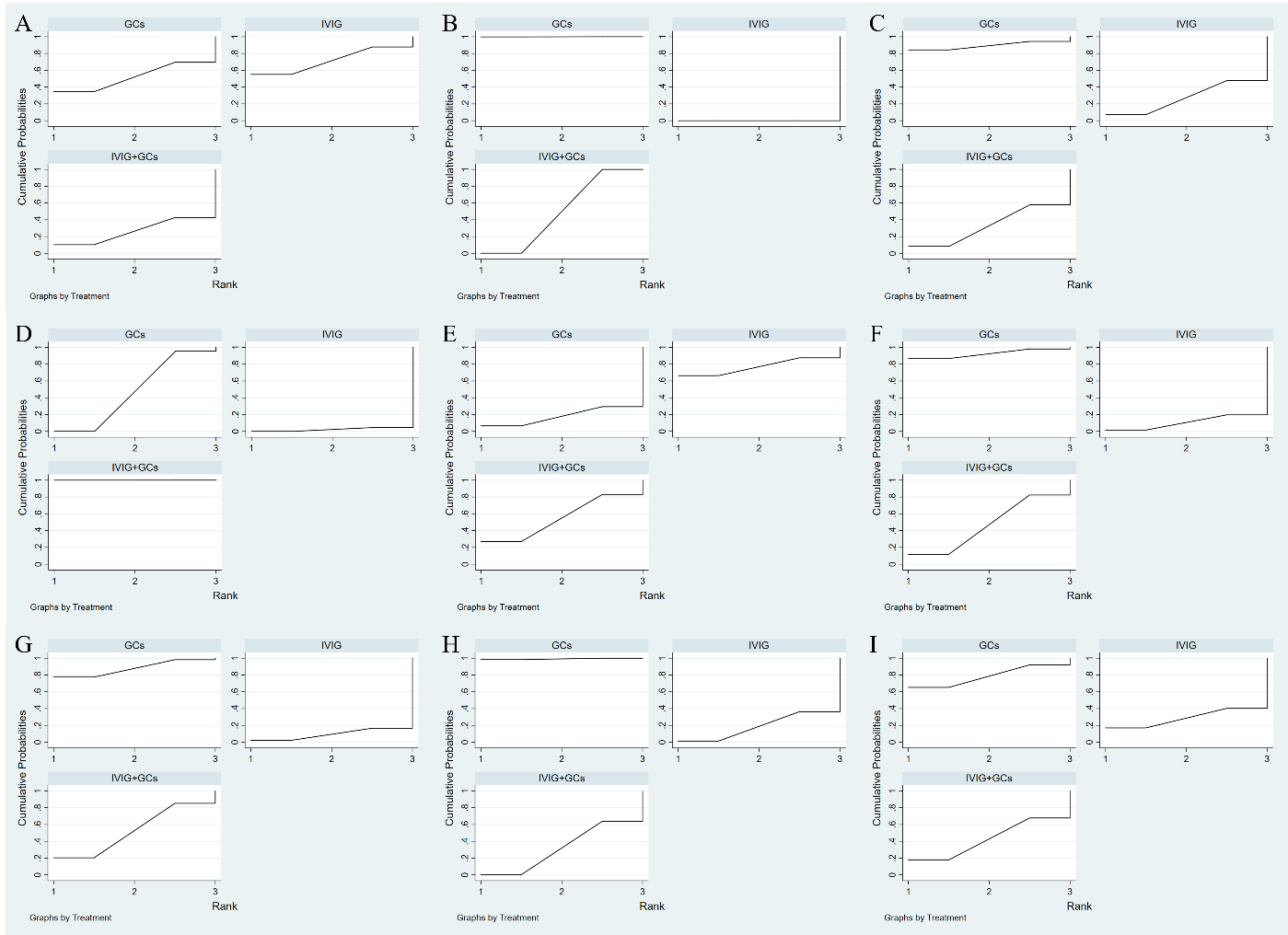


SUCRA graphs. Cumulative probability indicates the ranking of efficacy on the following: Adjuvant immunotherapy (A) , Persistent fever / failure of treatment (B), Need inotropic support (C), Left heart dysfunction (D), Mortality (E), Coronary Artery Dilatation (F), ICU long of stay (G), Duration of fever (H),Duration of inotropic support (I). The larger the surface under the curve, the better the rank of the intervention being the stipulation.

sFigure3.Funnel plot.


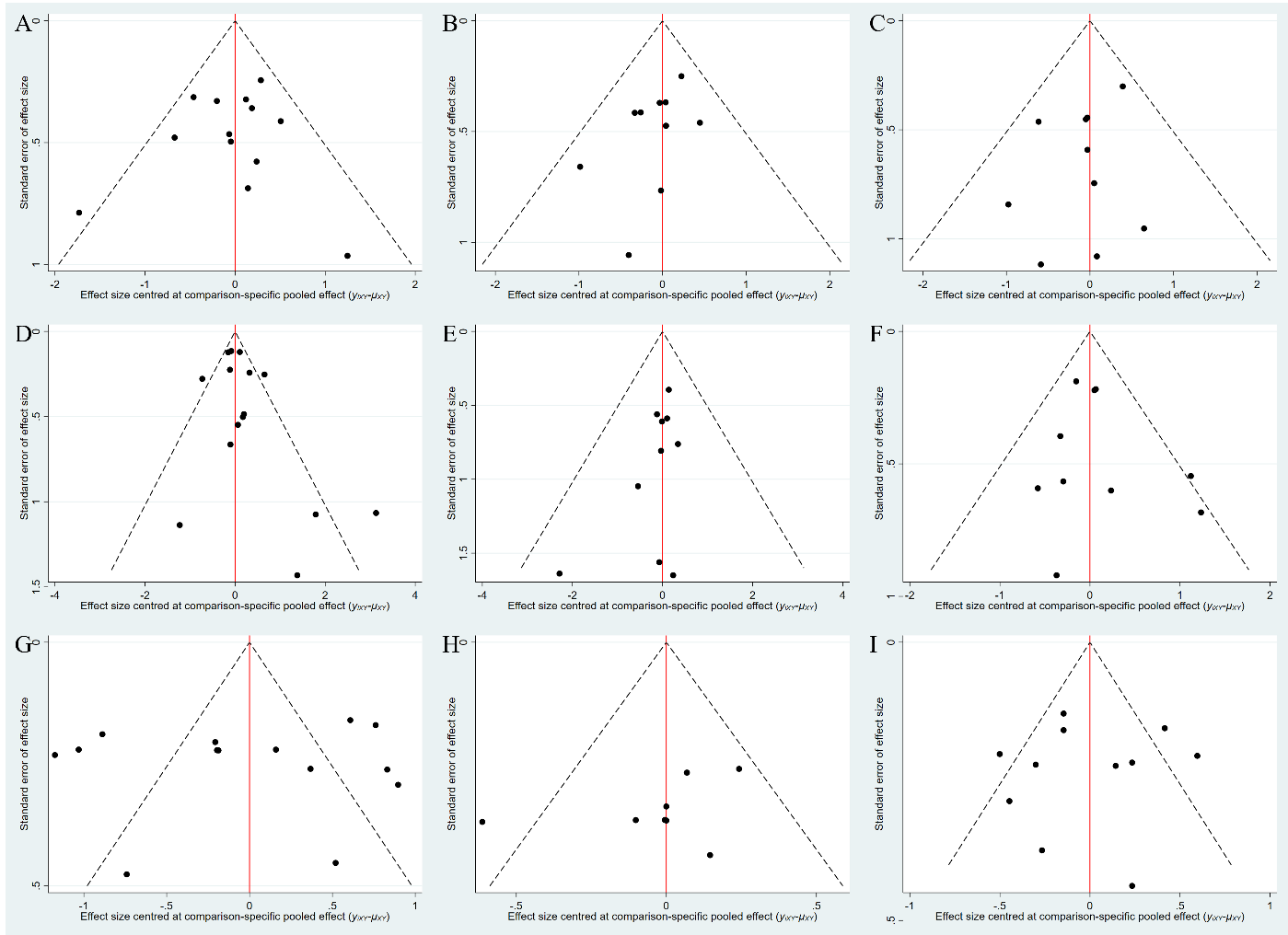


Funnel plot. Shown are funnel plot of network meta-analysis of all studies for Adjuvant immunotherapy (A) , Persistent fever / failure of treatment (B), Need inotropic support (C), Left heart dysfunction (D), Mortality (E), Coronary Artery Dilatation (F), ICU long of stay (G), Duration of fever (H),Duration of inotropic support (I).

sFigure4.Conformity Test.


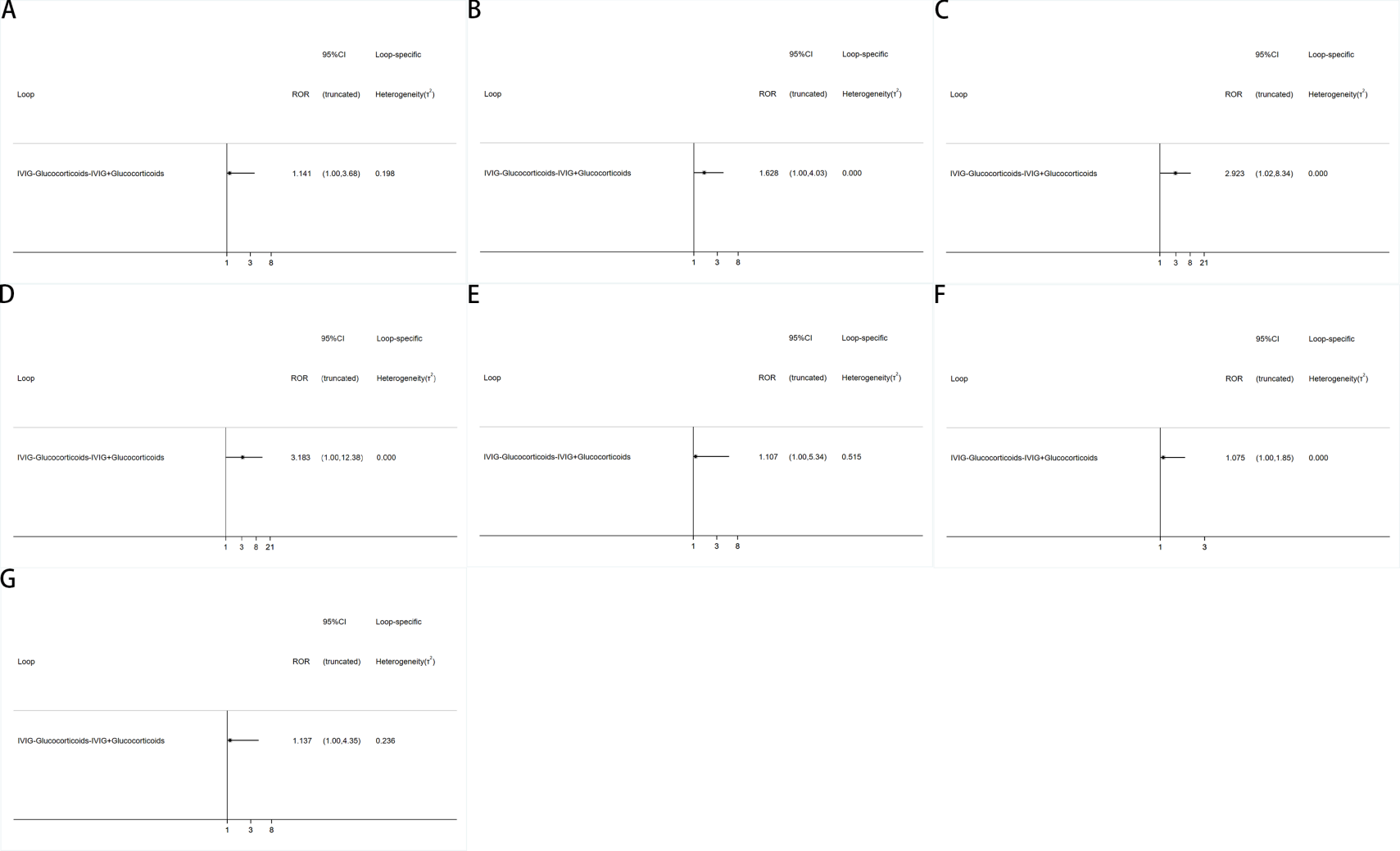


Conformity Test. Shown are conformity test of network meta-analysis of all studies for Adjuvant immunotherapy (A) , Persistent fever / failure of treatment (B), Need inotropic support (C) ,Left heart dysfunction (D),ICU long of stay (E),Duration of fever (F),Duration of inotropic support (G).
